# Food Ice Hygienic Quality Investigation from Public and Collective Catering

**DOI:** 10.3390/foods14071146

**Published:** 2025-03-26

**Authors:** Giuseppina Caggiano, Giusy Diella, Vincenzo Marcotrigiano, Paolo Trerotoli, Piersaverio Marzocca, Nicoletta De Vietro, Jolanda Palmisani, Alessia Di Gilio, Carlo Zambonin, Gianluigi De Gennaro, Giovanna Mancini, Antonella Maria Aresta, Letizia Lorusso, Anna Maria Spagnolo, Giovanni Trifone Sorrenti, Michele Lampedecchia, Domenico Pio Sorrenti, Ezio D’Aniello, Matilde Gramegna, Alessandra Nencha, Antonio Caputo, Marta Giovine, Caterina Spinelli, Francesco Triggiano

**Affiliations:** 1 Interdisciplinary Department of Medicine, Hygiene Section, University of Bari Aldo Moro, Piazza G. Cesare 11, 70124 Bari, Italy; giuseppina.caggiano@uniba.it (G.C.); giusy.diella@uniba.it (G.D.); paolo.trerotoli@uniba.it (P.T.); letizia.lorusso@uniba.it (L.L.); 2 Prevention Department, Local Health Authority “ULSS 1 Dolomiti”, Viale Europa 22, 32100 Belluno, Italy; vincenzo.marcotrigiano@aulss1.veneto.it; 3 Prevention Department, Food Hygiene and Nutrition Service Bari–North Area, Via De Chirico 23, 70056 Bari, Italy; piersaverio.marzocca@asl.bari.it (P.M.); antonio.caputo@asl.bari.it (A.C.); marta.giovine@asl.bari.it (M.G.); caterina.spinelli@asl.bari.it (C.S.); 4 Department of Biosciences, Biotechnologies and Environment, University of Bari Aldo Moro, Via Orabona 4, 70126 Bari, Italy; jolanda.palmisani@uniba.it (J.P.); alessia.digilio@uniba.it (A.D.G.); gianluigi.degennaro@uniba.it (G.D.G.); g.mancini1@studenti.uniba.it (G.M.); antonellamaria.aresta@uniba.it (A.M.A.); 5 Department of Health Sciences, University of Genova, Via Pastore 1, 16132 Genova, Italy; am.spagnolo@unige.it; 6 Prevention Department, Food Hygiene and Nutrition Service, Local Health Unit BT, 76125 Trani, Italy; giovannitrifone.sorrenti@uniba.it (G.T.S.); michele.lampedecchia@asl.bat.it (M.L.); domenicosorrenti@outlook.it (D.P.S.); 7 Prevention Department, Food Hygiene and Nutrition Service, Bari–Metropolitan Area, Piazza Chiurlia 21, 70122 Bari, Italy; ezio.daniello@asl.bari.it (E.D.); matilde.gramegna@asl.bari.it (M.G.); alessandra.nencha@asl.bari.it (A.N.)

**Keywords:** ice cube, water, public health, public and collective catering

## Abstract

In recent years, the global demand for food ice cubes has increased. The aim of the study was to evaluate the hygienic quality of both ice and water used for its production. During January–October 2023, 108 ice and water samples were collected in catering locations in Apulia Region and examined for *Escherichia coli*, Enterococci, total bacterial count (TBC) and fungi. Median counts of *E. coli*, Coliforms and Enterococci were 0 CFU/100 mL both for ice and water samples, collected in bars (*n* = 78) and restaurants (*n* = 30). The median TBCs in ice and water samples were 175 and 43 CFU/mL (*p* < 0.0001) at 22 °C, and 80 and 30 CFU/mL (*p* < 0.0001) at 36 °C. Total fungi counts were 4 and 0 CFU/mL for ice and water (*p* < 0.0001). In restaurants, differences were found between ice and water only for fungal contamination, whereas for bars, a difference was found between ice and water for Coliforms, Enterococci, TBC at 22 °C and fungi. The only statistically significant difference between bars and restaurants was observed for the TBCs at 22 °C (*p* = 0.017) and 36 °C (*p* = 0.036). Ice contamination does not appear to be directly related to the hygienic quality of water, but likely linked to the production, storage and maintenance of ice machines.

## 1. Introduction

“Food ice” is a product obtained directly from drinking water by freezing it, usually in cube form, to be consumed [1]. In recent years, the global demand for ice cubes intended for human consumption has increased [2,3]. In this context, there has been a proportional expansion in the number of food ice factories, although bars, pubs and restaurants also self-produce significant quantities of ice cubes through the use of production machinery [2,3].

Ice is increasingly used as an ingredient, edible ice, as a beverage cooler and for food storage [4,5]. Fresh foods come into direct or indirect contact with ice, such as raw fish, which is often stored in contact with a layer of ice prior to sale. Fruit and vegetables (e.g., strawberries and lettuce) are also often kept in direct contact with ice before eating, to ensure freshness [4].

A large number of bacteria can be found in ice, especially if poor quality water is used in its production. Therefore, beverages and food matrices that come into contact with ice are contaminated with the same pathogenic microorganisms [6,7]. Several studies have shown that many microorganisms can be found in food ice, mainly enteric bacteria [8,9] but also yeasts and filamentous fungi [2].

The quality and safety of packaged food ice, which does not meet the hygiene standards required for drinking water [1,10], are not only due to the use of contaminated water in ice production, but also to improper handling during production and [1,11] post-production contamination from processing and packaging [1,5,12].

Freezing cannot eliminate microorganisms, as so many of them can survive and become viable again upon thawing, causing foodborne diseases in consumers [1,13]. Outbreaks of foodborne illness directly or indirectly linked to contaminated ice have been reported in the literature [4,14,15].

Once melted, edible ice becomes potable water and must meet all food hygiene requirements [1]. A previous recent study [16] evaluated the hygienic quality of food ice cubes from public and institutional catering establishments in southern Italy, assessing their compliance with the legislation in force in Italy at the time (D.Lgs. 31/01) [17]. The authors detected the presence of *E. coli*, coliforms, *P. aeruginosa*, *S. aureus* and fungi.

On the basis of this findings, the aim of this study was to evaluate the hygienic quality of both the ice and the water used for its production, coming from the water supply network directly connected to the ice-making machines in accordance with the current and recent Legislative Decree 18/2023 [18] in force in Italy. Specifically, the authors wanted to assess whether any contamination of edible ice could be related to the water source or to the poor hygienic quality and maintenance of the ice-making machines.

## 2. Materials and Methods

### 2.1. Study Design

During the period from January to October 2023, 108 ice samples were collected directly from the storage compartments of the ice machines with notified activities of public and institutional catering establishments (bars and restaurants) located in two different areas of Apulia Region (southern Italy). For each food business, 1 kg of ice cubes was sampled using sterile tweezers and stored in sterile food bags.

Contextually, 1 litre of cold water (108 samples) was collected in sterile containers containing sodium thiosulphate pentahydrate (0.01%, *w*/*v*) to neutralise chloride present in the water. Samples were taken directly from the tap closest to the ice machine, eliminating any plastic components and fragiget screens; preliminary flaming, or, if not possible, chemically disinfecting the sampling site using 10% sodium hypochlorite; and subsequently proceeding with the water flushing for at least two minutes before performing the sampling, avoiding any change in the flow rate during the filling phase.

All ice and water samples were taken in the food establishments during the normal business activities. All the latter were connected to the public drinking water supply. The samples were immediately transported in isothermal containers to the Laboratory of Environmental and Food Hygiene of the University of Bari Aldo Moro, where they were processed within 24 h of sampling.

### 2.2. Microbiological Methods

Molten ice and water samples were processed to assess compliance with the minimum parameters (*Escherichia coli* and Enterococci) required by Legislative Decree 18/2023 [18]. Each sample was filtered through a 47 mm diameter cellulose ester membrane with a pore size of 0.45 μm (Millipore, Milan, Italy). Samples were considered acceptable if 100 mL of each sample was free of *E. coli* and Enterococci.

In addition, the total bacterial count (TBC) at 22 °C and 36 °C, coliforms, enumeration and detection of fungi were evaluated.

To detect *E. coli*, 100 mL of melted ice and water was filtered, and each membrane was placed on plates containing Chromogenic Coliform agar (Biolife Italiana Srl, Milan, Italy) and incubated at 36 ± 2 °C for 24 ± 2 h. The search for *E. coli* was performed according to UNI EN ISO 9308-1, 2017 [19], which also includes the search for coliforms. *E. coli* appeared as blue-violet colonies on culture media, while coliforms appeared as salmon-pink colonies and negative to the oxidase test [19]. Characteristic colonies were counted and the results expressed as Colony Forming Units (CFU)/100 mL.

However, to search for Enterococci, a further 100 mL aliquot of melted ice and water was filtered, and the membranes were then plated on Slanetz and Bartley agar (Biolife Italiana Srl) and incubated at 36 ± 2 °C for 48 h. If dark pink-red colonies were present, the membrane was transferred to Bile Esculin Azide agar (Biolife Italiana Srl) and incubated at 44 ± 0.5 °C for 2 h. Typical enterococcal colonies were identified by the presence of brown colonies with a brown-black halo and positive catalytic activity (UNI EN ISO 7899-2, 2003) [20]. The result was expressed as CFU/100 mL.

For the TBC, an aliquot of 1 mL of water and 1 mL of melted ice was taken from each sample, transferred to Petri dishes with Yeast Extract agar (Biolife Italiana Srl) and incubated at 36 ± 2 °C for 48 h and at 22 ± 2 °C for 72 h. The colonies grown on each plate were counted and the results expressed as CFU/mL (UNI EN ISO 6222, 2001) [21].

In addition, to detect other microorganisms not covered by the reference Legislative Decree 18/2023, 250 mL of each water and ice sample was filtered and each membrane was placed on PCA agar plates (Biolife Italiana Srl). The plates were incubated at 36 ± 2 °C for 48 h, and subsequently the microorganisms were identified by matrix-assisted laser desorption/ionisation time-of-flight mass spectrometry (MALDI-TOF MS, Biomèrieux, Marcy l’Etoile, Lyon, France).

As regards fungi, 100 mL of melted ice and 100 mL of water were filtered, then the membranes were placed on Sabouraud dextrose agar supplemented with chloramphenicol (0.5 g/L) (Liofilchem, Roseto degli Abruzzi, Italy), and incubated at 28 ± 2 °C for 10 days and the results were expressed as CFU/100 mL. The yeasts were identified by MALDI-TOF MS, while the filamentous fungi were identified by the evaluation of their macroscopic and microscopic morphological characteristics, as previously described according to the methods described by De Hoog [16,22].

### 2.3. Statistical Analysis

Bacterial and fungal counts were summarised as median and range as they did not follow a normal distribution. Comparisons were performed by non-parametric tests for independent groups (Wilcoxon–Mann–Witney) to assess the differences between bars and restaurants, and for paired groups (Wilcoxon sign ranks) to assess the differences between water and ice samples.

The analysis was also performed comparing percentages. Data were summarised as frequencies and percentages of samples with a value of CFU equal to 0 (zero).

The analysis of the proportions of the 0-count sample of each single bacterial/fungal pathogen was performed using the Fisher exact test (comparison between bar and restaurant) with an adjustment of the *p*-value accounting for the multiplicity of the comparisons, so a permutational method was applied to avoid false rejections of the null hypothesis. The adjustment of the *p*-values for paired groups was applied by a permutational method to the raw *p*-values resulting from the McNemar test (comparison between water and ice). Differences were considered statistically significant when the *p*-value was <0.05.

All analyses were performed using SAS 9.4 for PC with the FERQ procedure and the EXACT and AGREE options as appropriate. A *p*-value adjustment was performed using the MULTTEST procedure.

## 3. Results

A total of 110 establishments were sampled, but two had incomplete data and were excluded from the study. An analysis was performed on 108 businesses, of which 78 were bars and 30 were restaurants. Median counts of *E. coli*, Coliforms and Enterococci were 0 CFU/100 mL (zero/100 mL) both for ice and water samples and for both bars and restaurants.

The median TBC at 22 °C was 175 CFU/mL (range 0–1300 CFU/mL) for ice samples and 43 CFU/mL (0–800 CFU/mL) for water samples, and the difference was statistically significant (*p* < 0.0001) (Table 1). Similar results were found for the TBC at 36 °C with 80 CFU/mL (range 0–1500 CFU/mL) and 30 CFU/mL (0–662 CFU/mL) for ice and water, respectively, and the difference was statistically significant (*p* < 0.0001) (Table 1). The percentage of samples with a 0-count CFU for *E. coli* was 93.6% (104/108) and 100% (108/108) for ice and water, respectively, with no statistically significant difference (*p* = 0.125) (Table 1). The 0-count samples for coliforms were 56.48% and 80.56% for ice and water, respectively, and the difference was statistically significant (*p* < 0.0001). The difference in the percentage of 0-count samples between ice and water was also statistically significant (*p* < 0.0001) for Enterococci with 80.55% and 99.07% for ice and water, respectively.

Total fungal counts (including yeasts and moulds) were 4 CFU/mL (range 0–150 CFU/100 mL) for ice and 0 CFU/mL (range 0–80 CFU/100 mL) for water, and the difference was statistically significant (*p* < 0.0001). The percentage of fungal 0-count samples was 8.33% for ice and 64.81% for water (Table 1), and the difference was statistically significant (*p* < 0.0001).

The median count of *E. coli* was zero in both bars and restaurants and in both ice and water, so the difference was not statistically significant (*p* = 0.125). For total coliforms and Enterococci, the median count was 0, but the distribution of counts showed a statistically significant difference between bars and restaurants; furthermore, there was a difference in microbial counts at 22 °C (*p* = 0.0004) and at 36 °C (*p* = 0.0092).

When comparing the results between ice and water in the bar subgroup, a statistically significant difference was found for the TBC at 22 °C (*p* < 0.0001) and TBC at 36 °C (*p* < 0.0001). The same comparisons in the restaurant’s subgroup showed no statistically significant difference for any bacterial/fungal parameter (*p* = 0.8073 and *p* = 0.6095, respectively).

The comparisons of the percentage of 0-count samples are shown in Table 2. In the restaurant subgroup, no difference between ice and water came out as statistically significant, with the exception of fungal contamination, which was lower in the water: the percentage of 0-counts between ice and water was 13.3% and 50%, respectively (*p* = 0.0127). A different condition was observed in the bar subgroup, where we found a statistically significant difference between ice and water for total coliforms (respectively, 56.4% vs. 79.5%, *p* = 0.0005), Enterococci (respectively, 76.9% vs. 97.4%, *p* < 0.0001), TBC at 22 °C (respectively, 2.6% vs. 15.4%, *p* = 0.0063) and fungi (7.7% vs. 67.9%, *p* < 0.0001). The CFU for *E. coli* was almost all zero in both bars and restaurants.

The only statistically significant difference between bars and restaurants was observed for the TBC at 22 °C and 36 °C: the first was 2.6% in bar ice and 16.7% in restaurant ice (*p* = 0.017); the second was 15.4% in bar water and 3.3% in restaurant water (*p* = 0.036).

Details of the isolated bacteria are shown in Table 3. The most frequent bacteria detected were *Staphylococcus epidermidis* and *Staphylococcus warnerii*, both in water (13.8% and 14.8%, respectively) and ice (24.07% and 22.22%, respectively), but the percentage of samples was not statistically different. *Delftia acidovorans* (12.96% in both water and ice samples) and *Micrococcus* spp. (11.8% in water and 11.11% on ice) followed as the most frequent microbial agents, but again the difference was not statistically significant.

In Table 4, the fungal analysis description is shown. Among the most frequently isolated yeasts, *Rhodutorula mucillaginosa* and *Candida guilliermondii* were found in ice, in 10.19% and 5.56% of ice samples, respectively, but not in water, where only *Rhodutorula mucillaginosa* was found in 1.85% of water samples.

For moulds in general, ice samples had significantly more species than water samples. In particular, ice samples showed a higher percentage of all *Aspergillus* species except *A. nidulans* compared to water (Table 4). The highest difference was for the *A. fumigatus*: it was found in 31.5% of ice and 6.5% of water samples.

Other moulds detected in a high percentage of samples were *Cladosporium* spp. and *Penicillium* spp. detected, respectively, in 31.5% of ice vs. 9.3% of water samples (*p* = 0.0001), and 19.4% of ice vs. 1.8% of water samples (*p* < 0.0001).

**Figure 1:**
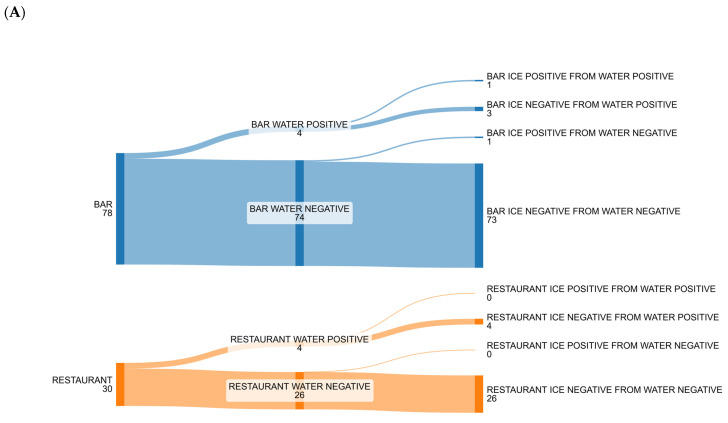

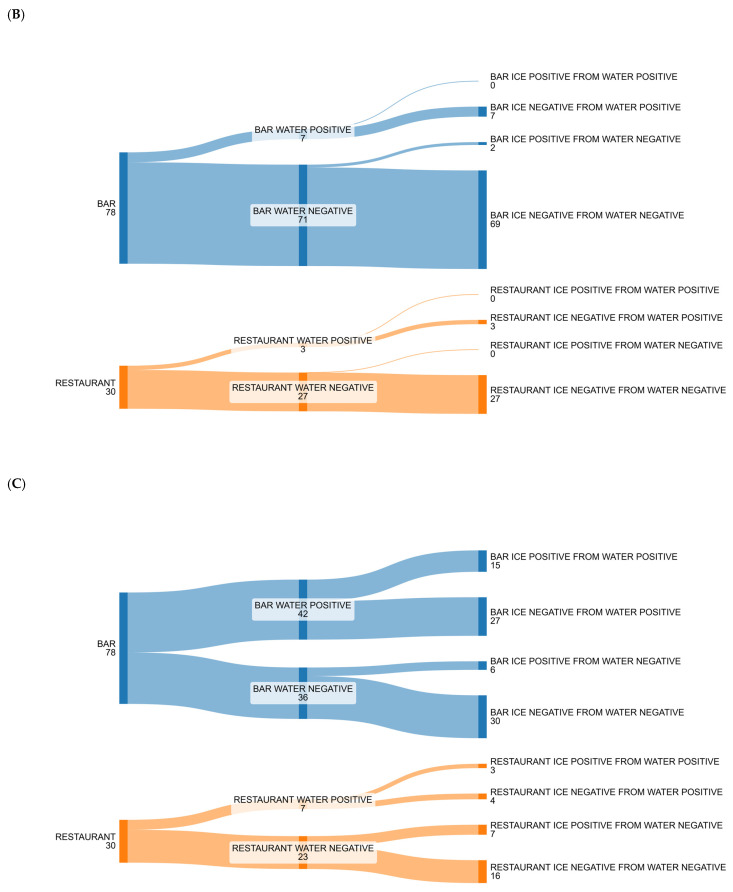
Alluvial plot to show the changeover of positive and negative results from water to ice by bars and restaurants of the most abundant isolated bacteria: (**A**) *Acinetobacter*: bars and restaurants had mostly negative water, that remained negative, while there were few cases of positive water samples and changes into negative ice; (**B**) *Enterobacter*: in both bars and restaurants there were a small number of positive water samples that changed into negative ice samples, but in bars there were also a small number of negative water samples that changed into positive ice samples; (**C**) *Staphylococcus* spp.: in bars there were a large number of positive water samples, but 63% of them changed into negative ice samples and a small number of negative water samples changed into positive ice samples; a similar condition could be observed in restaurants’ water and ice samples.

**Figure 2:**
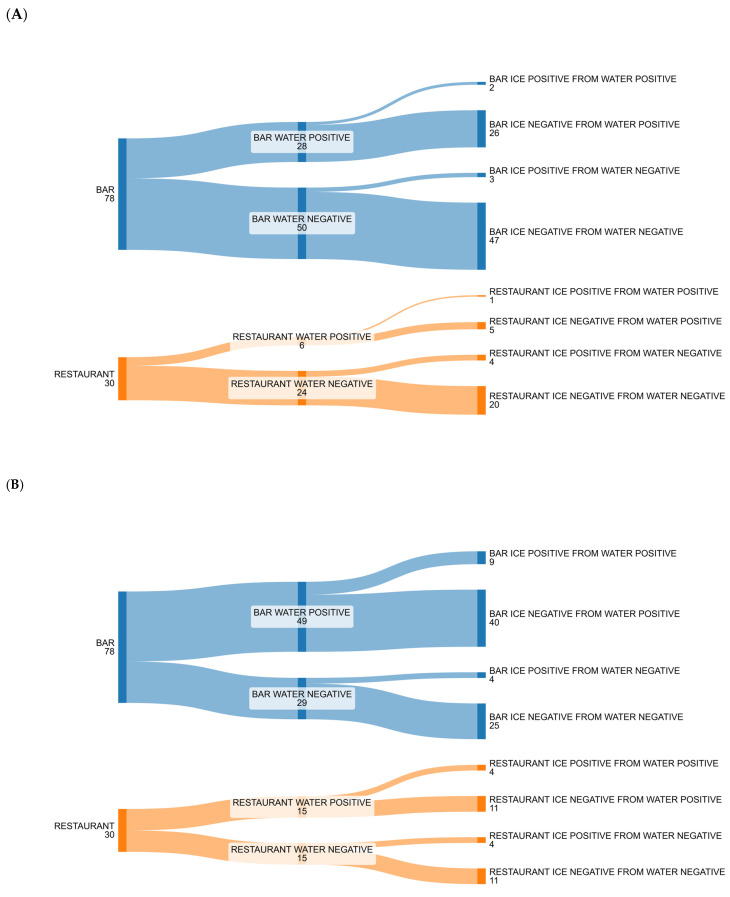
Alluvial plot to show the changeover of positive and negative results from water to ice by bars and restaurants of the most abundant isolated fungi: (**A**) *Cladosporium* spp.: in both bars and restaurants there were a small number of positive water samples that changed into negative ice samples, but in bars there were also a small number of negative water samples that changed into positive ice samples; a similar condition could be observed in restaurants’ water and ice samples; (**B**) *Aspergillus* spp.: in bars there were a large number of positive water samples, but a lot of them changed into negative ice samples, while a small number of negative water samples changed into positive ice samples; a similar condition could be observed in restaurants’ water and ice samples.

Figure 1 and Figure 2 show the number of positive and negative samples for the most commonly isolated microorganisms (bacteria and fungi) in relation to the type of matrix (both water and ice) and different activities (both bars and restaurants).

## 4. Discussion

There are only a few studies in the literature that have evaluated the contamination of food ice in the world. Despite this evidence, all studies have shown that ice cannot currently be considered a safe food, since it contains bacteria, viruses and fungi [23].

To date, there is no ad hoc regulation in Italy aimed at carrying out controls on food ice, by defining both the frequency of controls and the parameters to be investigated. Therefore, since melted food ice becomes water, reference is made to Legislative Decree 18/23 [18]. In this regard, in our study, 25 (23.14%) ice samples were non-compliant for the presence of *E.coli* and Enterococci, and one ice sample was non-compliant only for the presence of Enterococci. In addition to these results, most of the water and ice samples were found to be contaminated with other microorganisms not covered by the reference standards. Among these, we found bacteria such as *Staphylococcus epidermidis*, *Staphylococcus warnerii*, *Bacillus cereus* group, *Acinetobacter* spp. and *Enterobacter* spp., microorganisms responsible for infectious complications especially in immunocompromised subjects, where these bacteria are able to colonise the mucous membranes of the oral cavity and the gastro-intestinal tract. On the other hand, among the fungi, we found moulds, including *Cladosporium*, *Aspergillus* spp. and *Penicillium* spp. and yeasts such as *Rhodutorula mucilaginosa*, *C. guilliermondii*, *C. lusitaniae* and *C. parapsilosis*. These microorganisms, although not classified as primary pathogens, can be potentially dangerous for susceptible individuals such as immunocompromised persons, children and the elderly who may consume this food matrix. In particular, yeasts such as *C. parapsilosis* are often responsible for invasive diseases, which also lead to nosocomial clusters often with difficult therapeutic management. The onset of invasive disease may be a consequence of colonisation of the fragile patients predisposed to such infectious complications [24]. In addition, *C. lusitaniae* is also genetically resistant to amphotericin B, considered the gold standard of antifungal therapy [25]. In addition, haematological patients, in particular, suffer from post-treatment mucositis, which can be a route of entry for potential pathogens as well as fungal spores of *Aspergillus* spp. These can lead to invasive forms of aspergillosis, bearing in mind that food ice is often recommended to relieve the pain associated with post-chemotherapy mucositis [16,26,27].

The presence of these bacteria and moulds could be related to improper maintenance and cleaning of ice machines and incorrect use of protective equipment (gloves) by food users. For example, if gloves are not properly used and changed, some microorganisms present on the skin, such as Staphylococci, may be transferred directly from operators’ hands to the ice and ice machine. In addition, poor maintenance of ice machines can lead to the colonisation of microorganisms in the pipes of the machine, which are consequently transferred to the ice.

Our results are similar to those of Hampikyan et al. [5], who evaluated the microbiological quality of ice and water used for ice production in different food establishments in Turkey (105 structures including restaurants/fast food, bars and fish markets). The analyses were aimed at detecting *E. coli*, coliforms, Enterococci and total aerobic psychrophilic and mesophilic bacteria. The results showed the presence of *E. coli*, Enterococci and psychrophilic bacteria in ice samples, while they were absent in all water samples analysed. Coliforms and total aerobic mesophilic bacteria were detected in both water and ice samples.

Contamination with *E. coli* and other pathogens (*Salmonella* spp. and *Yersinia* spp.) has also been reported in other studies on ice cubes [6,7]. The presence of fungi in ice cubes has also been highlighted in the studies conducted in Italy by Francesca et al. [2] and Caggiano et al. [16].

Several studies in the literature have shown how the consumption of contaminated ice can be a direct or indirect route for the transmission of pathogenic bacteria to humans and consequently lead to outbreaks of gastro-intestinal diseases [5,28].

Research into the microbial quality of both water and ice concluded that the water supply was not the primary source of contamination, and that the transfer of bacteria to the ice was due to the irregular cleaning of the water tank and freezer and the lack of cleaning and maintenance [5].

In order to prevent the spread of microorganisms and contaminants in ice-making machines, food business operators (FBOs) are required to implement preventive measures, such as regular and proper cleaning and maintenance of the machines, and, where considered a critical control point, sampling of the ice matrix and the water distributed in food business activities. As part of the plans based on the principles of the HACCP System, FBOs must carry out a prior assessment and define the measures for monitoring the parameters considered essential for control (Reg. EC 852/04) [29]. Proper monitoring, carried out with sufficient frequency, can help to detect any non-compliance early. Furthermore, the frequency of the FBO’s own checks must take into account the origin of the water: usually, if it comes from the public aqueduct, it provides greater guarantees. On the other hand, if the food business uses water from different supplies (e.g., from a well, rainwater recovery, spring, etc.), an in-depth assessment and monitoring must be carried out, especially if there is an independent water purification system and a storage tank that must be kept in good working order. Moreover, the presence of any other critical point in the water distribution system (i.e., filters, softeners, resins, etc.) must be carefully assessed using a risk-based approach. To the best of our knowledge, this is the first study in our country to simultaneously investigate the microbiological quality of both the water used for ice production and the ice cubes. In the future, the quality of cleaning of the internal surfaces of the ice machines could also be evaluated, in order to possibly correlate the presence of bacteria and fungi in the ice samples tested. Furthermore, the concentration of free residual chlorine was not measured in the sampled drinking water, on the assumption that the aqueduct managing authority that supplies the local structures ensures adequate chlorine coverage there.

## 5. Conclusions

In light of the evidence that has emerged, ice contamination does not appear to be directly related to the hygienic quality of the water matrix, but to the production process and storage in ice-making machines, including their maintenance. In view of the findings reported here, the methods of use, proper maintenance and disinfection and frequency of checks on the equipment and ice need to be effectively regulated by the competent local and national authorities. Finally, the training of all FBOs represents an important prerequisite for the safe production, storage and administration of ice intended for human consumption.

## Figures and Tables

**Table 1:** Microbial counts at 22 °C and 36 °C, and percentage of 0-count samples for *E. Coli,* Coliforms and Enterococci in ice and water samples.

Microorganisms	Samples				*p*-Value
	Ice Samples		Water Samples		
	175	0–1300	43	0–800	<0.0001 *
Total bacterial count at 36 °C (median, range)	80	0–1500	30	0–662	<0.0001 *
*E. coli* (No., %)	104	96.3	108	100	0.1250 ^§^
Coliforms (No., %)	61	56.48	87	80.56	<0.0001 ^§^
Enterococci (No., %)	87	80.55	107	99.07	<0.0001 ^§^
Fungi (No., %)	9	8.33	70	64.81	<0.0001 ^§^

Legend: *: Wilcoxon for paired data; ^§^: McNemar test for paired proportion.

**Table 2:** Frequency and percentage of sample with 0 (zero) CFU for each microbial test and establishments and ice/water samples.

Microorganisms	Bar (No. = 78)				Restaurants (No. = 30)			
	Ice Samples		Water Samples		Ice Samples		Water Samples	
	No.	%	No.	%	No.	%	No.	%
*E. coli*	73	93.6	76	97.4	30	100.0	30	100.0
Coliforms	44	56.4 ^c^	62	79.5 ^c^	17	56.7	23	76.7
Enterococci	60	76.9 ^d^	76	97.4 ^d^	25	83.3	29	96.7
Count at 22 °C	2	2.6 ^ae^	12	15.4 ^e^	5	16.7 ^a^	1	3.3
Count at 36 °C	9	11.5	15	19.2 ^b^	3	10.0	1	3.3 ^b^
Fungi	6	7.7 ^f^	53	67.9 ^f^	4	13.3 ^g^	15	50.0 ^g^

*p*-values statistically significant: ^a^: 0.017; ^b^: 0.036; ^c^: 0.0005; ^d^: <0.0001; ^e^: 0.0063; ^f^: <0.0001; ^g^: 0.0127.

**Table 3:** Percentage of positive samples for each bacterium in ice and water samples.

Microorganisms	Ice Samples (No. = 108)		Water Samples (No. = 108)		*p*-Value
	No.	%	No.	%	
*Acinetobacter baumannii*	1	0.93	0	0.0	1.0
*Acinetobacter gyllenbergii*	7	6.48	2	1.85	0.0588
*Acinetobacter jejuni*	10	9.25	8	7.41	0.4795
*Acinetobacter ursingii*	6	5.55	2	1.85	0.1025
*Bacillus cereus group*	23	21.3	31	28.70	0.1824
*Delftia acidovorans*	14	12.96	14	12.96	1.0
*Enterobacter* spp.	10	9.09	2	1.82	0.0386
*Klebsiella oxytoca*	4	3.7	2	1.85	0.3173
*Klebsiella pneumoniae*	4	3.7	1	0.93	0.1797
*Lelliotta amnigena*	3	2.78	1	0.93	0.3173
*Micrococcus* spp.	12	11.11	13	11.82	1.0
*Pseudomonas aeruginosa*	7	6.48	6	5.56	0.7630
*Pseudomonas putida*	4	3.7	3	2.78	0.7055
*Staphylococcus epidermidis*	25	24.07	15	13.89	0.0482
*Staphylococcus warnerii*	24	22.22	16	14.81	0.1306

**Table 4:** Comparison of percentage of positive samples between ice and water for each yeast/mould.

	Ice Samples (No. = 108)		Water Samples (No. = 108)		*p*-Value
Yeasts	No.	%	No.	%	
*Exophiala* spp.	4	3.7	3	2.8	1
*C. albicans*	3	2.78	0	0.0	0.25
*C. guilliermondii*	6	5.56	0	0.0	0.0313
*C. lusitaniae*	3	2.78	0	0.0	0.25
*C. parapsilosis*	3	2.78	0	0.0	0.25
*C. fusiformata*	1	0.93	0	0.0	1.0
*Cryptococcus curvatus*	1	0.93	0	0.0	1.0
*Rhodutorula mucillaginosa*	11	10.19	2	1.85	0.0225
**Moulds**					
*Absidia* spp.	1	0.93	2	1.85	1.0
*A. candidus*	19	17.59	4	3.7	0.0015
*A. flavus*	7	6.48	0	0.0	0.0156
*A. fumigatus*	34	31.48	7	6.48	<0.0001
*A. nidulans*	2	1.85	4	3.7	0.625
*A. niger*	18	16.67	3	2.78	0.0003
*A. terreus*	14	12.96	6	5.56	0.0215
*Alternaria* spp.	1	0.93	2	1.85	1.0
*Chrysosporium* spp.	4	3.7	1	0.93	0.3750
*Cladosporium* spp.	34	31.5	10	9.26	0.0001
*Fusarium* spp.	0	0.0	1	0.93	1.0
Mucorales	6	5.56	1	0.93	0.125
*Penicillium* spp.	21	19.44	2	1.85	<0.0001
*Scopulariosis* spp.	1	0.93	0	0.0	1.0
*Trichopyton* spp.	3	2.78	3	2.78	1.0

## Data Availability

The original contributions presented in the study are included in the article and further inquiries can be directed to the corresponding authors.

## Abbreviations

The following abbreviations are used in this manuscript:

CFU	colony forming units
FBOs	Food business operators
MALDI-TOF MS	matrix-assisted laser desorption/ionisation time-of-flight mass spectrometry
TBC	Total bacterial count
